# Insights into the Mechanisms of Action of Proanthocyanidins and Anthocyanins in the Treatment of Nicotine-Induced Non-Small Cell Lung Cancer

**DOI:** 10.3390/ijms23147905

**Published:** 2022-07-18

**Authors:** Naser A. Alsharairi

**Affiliations:** Heart, Mind & Body Research Group, Griffith University, Gold Coast, QLD 4122, Australia; naser.alsharairi@gmail.com

**Keywords:** flavonoids, proanthocyanidins, anthocyanins, nicotine, NSCLC

## Abstract

In traditional medicine, different parts of plants, including fruits, have been used for their anti-inflammatory and anti-oxidative properties. Plant-based foods, such as fruits, seeds and vegetables, are used for therapeutic purposes due to the presence of flavonoid compounds. Proanthocyanidins (PCs) and anthocyanins (ACNs) are the major distributed flavonoid pigments in plants, which have therapeutic potential against certain chronic diseases. PCs and ACNs derived from plant-based foods and/or medicinal plants at different nontoxic concentrations have shown anti-non-small cell lung cancer (NSCLC) activity in vitro/in vivo models through inhibiting proliferation, invasion/migration, metastasis and angiogenesis and by activating apoptosis/autophagy-related mechanisms. However, the potential mechanisms by which these compounds exert efficacy against nicotine-induced NSCLC are not fully understood. Thus, this review aims to gain insights into the mechanisms of action and therapeutic potential of PCs and ACNs in nicotine-induced NSCLC.

## 1. Introduction

**Figure 1:**
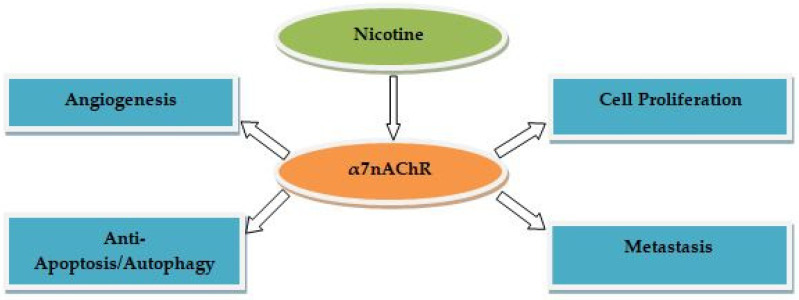
Mechanisms for nicotine in the development of NSCLC.

Lung cancer (LC) is considered the main diagnosed cancer causing death worldwide [1]. LC is broadly categorized into two major histologic classes: small cell lung cancer (SCLC) and non-small cell lung cancer (NSCLC). NSCLC is further subclassified into squamous, adenocarcinoma and large cell carcinoma, which constitute 85% of smoking-attributable LC cases [2,3]. Tobacco products contain several carcinogenic compounds, such as tobacco-specific nitrosamines (i.e., NNN and NNK), which enhance production of DNA adducts in the lungs of smokers, thereby causing mutations of several NSCLC suppressor genes including protein p53 [4,5,6]. Although nicotine is considered non-carcinogenic, it may contribute to NSCLC development [7,8,9,10,11]. Nicotine enhances proliferation, angiogenesis and metastasis and inhibits apoptosis/autophagy in NSCLC cells by activating nicotinic acetylcholine receptors (nAChRs), especially the α7 subunit, and its downstream signaling pathways including the proto-oncogene serine/threonine kinase (Rb-RAF1), the phosphatidylinositol-3 kinase/serine/threonine kinase (PI3K/Akt), the mammalian target of rapamycin (mTOR), the nonreceptor tyrosine/kinase focal adhesion/protein kinase (Src/FAK/PKC) and the extracellular signal-regulated kinase/mitogen-activated protein kinase (ERK/MAPK) [12]. Nicotine also induces epithelial-to-mesenchymal transition (EMT), which is the key step in enhancing tumor progression in NSCLC cells, resulting in upregulation of several transcription and growth factors via activation of α7nAChR-mediated signaling pathways [12]. The mechanisms by which nicotine enhances tumor progression in NSCLC cells have been previously described in greater details [13]. In brief, nicotine binds to α7nAChR, activating the cellular signaling pathways involved in proliferation, metastasis, angiogenesis and anti-apoptosis/autophagy, which increases expression of EMT-associated molecules in NSCLC cells such as hypoxia inducible factor-1 (HIF-1α), vascular endothelial growth factor (VEGF), transforming growth factor β (TGF-β), deca-pentaplegic homolog (Smad), B-cell lymphoma-2 (Bcl-2), metalloproteinases (MMPs), cyclinD1, Snail, twist, vimentin, fibronectin and N-cadherin [13]. Figure 1 summarizes the mechanisms for nicotine in the development of NSCLC.

There is no clear recommendations on safe dietary supplements for use in treating NSCLC, particularly in smokers [14]. However, combination therapy of monoclonal antibody-based immunotherapy and chemotherapeutic agents has been shown to be a promising treatment strategy for NSCLC [15]. In addition, natural flavonoids present in fruits (e.g., citrus) in combination with chemotherapeutic agents, such as cisplatin, have shown anti-NSCLC effects, demonstrated by inhibition of the α7nAChR-mediated signaling pathways involved in cellular processes including proliferation, inflammation and anti-apoptosis [13,16].The health benefits of fruits are due to the high levels of bioactive flavonoids they contain, such as PCs and ACNs [17,18]. A large number of studies have documented the health benefits of natural flavonoids derived from fruits and plant foods, in relation to their biological attributes such as anti-diabetes/anti-cancer activity, reducing cardiovascular diseases and improving the blood lipid profile [17,18,19]. Thus, there is a real need to investigate natural flavonoid compounds, such as PCs and ACNs, as therapeutic agents for nicotine-induced NSCLC.

Flavanols include a group of natural compounds categorized according to their chemical structures into catechins and PCs, which are found in various plant-based foods. PCs, also known as condensed tannins, are polymeric and/or oligomeric pigments found in common plant-based foods (e.g., fruits, vegetables, cereal grains, and legumes), Cinnamomi Cortex (barks of *Cinnamomum cassia* used as a traditional Chinese medicine) and *Vaccinium* berries, for which a range of therapeutic effects have been reported including anti-cancer, antimicrobial, anti-diabetic, anti-obesity, cardioprotective and antioxidant properties [20]. Procyanidins are the most homo-oligomeric PC derivatives comprised of epicatechin/catechin monomeric, connected via the C4→C6/C4→C8 bond (B-type linkage) and the C2→O7 bond (A-type linkage), and dominated by dimers (e.g., procyanidin A1-A2/B1-B8), trimers (e.g., selligueain A/B and procyanidin C1/C2), and tetramers (degree of polymerization ranged from 5 to 11) [20,21,22].

ACNs, featuring six common glycosylated forms of anthocyanidins (i.e., malvidin, pelargonidin, cyanidin, petunidin, delphinidin and peonidin) with hydroxyl (OH) moieties in their structure at the 3 position on the C-ring are water-soluble pigments belonging to flavonoids responsible for producing various colors in fruits, berries and vegetables that exert a protective effect against diabetes, cardiovascular and neurodegenerative diseases and cancers (including LC) [23,24,25].

In various plant species, PCs and ACNs share the first and last steps of flavonoid biosynthesis and its regulatory genes including dihydroflavonol reductase, flavanone 3β-hydroxylase and chalcone isomerase/synthase [26,27]. PCs and ACNs haveshown to be the key compounds contributing to the intense color of cereals, fruits, vegetables and blueberries [28], and they have been reported to reduce the production of reactive oxygen species (ROS) in some berry fruits, which may make them potential anti-antioxidant agents [29]. Development and ripening of plant species involve complex processes that result in substantial structural and metabolic changes such as changes in texture and flavor formation and pigment accumulation [30]. These processes are coordinated by abscisic acid (ABA) and other ripening-related genes, which regulate PCs and ACNs biosynthesis [30]. Ripening is considered an important stage where major flavonoids are accumulated [31]. A diverse range of plant phytohormones including cytokinin, gibberellins, auxin, jasmonic acid and brassinosteroids are involved in regulating different aspects of fruit development and ripening through multilevel regulatory mechanisms [31]. PCs are the main compounds detected at the early stage of plant development, whereas ACNs accumulate early in ripening [31]. ACNs act as the major colored pigments attracting animals and insects for seed dispersal in berry fruits [30]. In climacteric fruit ripening, ethylene plays a key hormonal role in stimulating the differential expression of many gene encoding transcription factors that regulate pigments accumulation, cell wall softening, texture change and aroma, flavor and skin color development [32,33].

A recent reviews showed that particular flavonoid compounds (e.g., quercetin, kaempferol, epigallocatechin gallate, and delphinidin) derived from plant-based foods act against NSCLCin in vitro/in vivo models [16,34]. However, the molecular mechanism of PCs and ACNs with regard to their preventive effects against nicotine-induced NSCLC have not been fully elucidated. A recent review showed that flavone-rich extracts from *Scutellaria baicalensis*, and baicalein, baicalin, wogonin, wogonoside and oroxylin A, in particular, serve as potential adjuvant agents for the treatment of nicotine-induced NSCLC. These compounds exert anti-inflammatory, anti-angiogenesis, anti-proliferative and apoptotic/autophagic effects through inhibiting several cellular signaling pathways as potential molecular mechanisms [13].

The therapeutic effects of PCs and ACNs against nicotine-induced NSCLC may depend on their bioavailability, that is, absorption, distribution and excretion. ACNs are distinguishable from other flavonoids because of its apparent low bioavailability [35,36]. However, studies have demonstrated that ACNs have higher bioavailability and bioactivity in vitro than in vivo [37]. ACNs are absorbed in the blood, distributed to target tissues and excreted into bile as the glycosylated structure [35]. The absorption efficiencies of the hydrophobic ACNs are greater than the hydrophilic ones in vitro [38]. In vitro studies showed that PAs oligomers are degraded into monomers and dimers in acidic gastric juice by human fecal microflora, which are then absorbed as intact molecules in the colon and excreted into urine. In vivo, epicatechin (EC) and methylated EC, as the major metabolites of PAs dimers, are absorbed in the intestinal mucosa in very low contents, catabolized by fecal microflora fermentation into low molecular phenolic acids and then excreted into urine [39]. Given that PCs and ACNs are used as therapeutic agents against many chronic diseases, targeting these compounds might help our understanding of the mechanisms involved in nicotine-induced NSCLC treatment, particularly in smokers. To date, no reviews have discussed the molecular mechanisms of PCs and ACNs in nicotine-induced NSCLC treatment. Thus, this paper highlights the mechanisms of action and therapeutic potential of PCs and ACNs in nicotine-induced NSCLC.

## 2. Methods

A literature review was undertaken to identify the published studies exploring the potential molecular mechanisms of PCs and ACNs in nicotine-induced NSCLC treatment from 2000 up to June 2022 via searches of PubMed/MEDLINE. The following search terms were used:“proanthocyanidins” OR “procyanidin” AND “anthocyanins” OR “malvidin” OR “pelargonidin” OR “cyanidin” OR “petunidin” OR “delphinidin” OR “peonidin” AND “NSCLC”OR “lung cancer” AND “nicotine” OR “cigarette smoke”. The search included all types of study design published in English. All studies were identified and reviewed by the author. From a total of 202 articles searched using the databases, 30 studies were selected based on the search terms.

## 3. Proanthocyanidins in Nicotine-Induced NSCLC Treatment

Several studies demonstrated the therapeutic effects of PC-rich extracts from plant-based foods and/or medicinal plants at different nontoxic concentration against nicotine-induced NSCLC. Cranberry-derived PCs suppress tumor cell growth in NSCLC cells [40,41], but the mechanisms for this action have not been well investigated [40]. Treatment with cranberry PCs resulted in a significant induction of apoptosis and cell cycle arrest in NSCLC cells via upregulating the expression of pro-apoptotic-related markers (e.g., cytochrome *c* and caspase 3) [42].

PCs from grape seed extract have shown promising results in nicotine-induced NSCLC treatment. For example, using the in vivo proteolysis/antitumor assay and the in vitro proteolysis/angiogenesis assay, PCs inhibit angiogenesis-mediated tumor growth in NSCLC cells, in part by suppressing vascular extracellular matrix (ECM) proteolysis byMMP-2 [43]. Treatment of NSCLC cells with PCs using the in vivo tumor xenograft assay and the in vitro 3-(4,5-dimethylthiazol-2-yl)-2,5-diphenyltetrazolium bromide (MTT) assay for cell proliferation/survival resulted in suppression of cell proliferation in vitro/vivo, and inhibition of angiogenesis and induction the apoptotic cell death of tumor cells in vivo. Such effects are mediated by upregulation of insulin-like growth factor binding protein-3 (IGFBP-3) levels and inhibition of proliferating cell nuclear antigen (PCNA) in the tumor microenvironment [44]. A study used the in vivo tumor xenograft assay and the in vitro cell deathenzyme-linked immunosorbent assay (ELISA) and MTT assay for assessing proliferation of NSCLC cells showed that PCs cause proliferation inhibition and apoptosis induction via the inhibition of cyclooxygenase-2 (COX-2) expression and prostaglandin-2 (PGI-2) receptors in NSCLC cells [45]. The mechanism underlying the anti-migration effect of PCs on NSCLC cells involve inhibiting nitric oxide (NO) synthase, N(G)-nitro-L-arginine methyl ester (L-NAME), and the ERK1/2 and MAPK signaling pathways [46]. The anti-proliferative/apoptotic effects of PCs onNSCLC cells are mediated via the activation of caspase 3 expression, prostacyclin synthase (PTGIS)/PGI2 (as measured by 6-keto PGF1α), and 15-lipoxigenase-2/15(S)-hydroxyeicosatetraenoic acid (15-LOX-2)/15-HETE production [47]. PCs showed the inhibitory effects on the cigarette smoke condensate (CSC)-induced migration of NSCLC cells through inhibition of NADPH oxidase (NOX)-induced oxidative stress and EMT transition [48]. Treatment with PCs using the colorimetric caspase-3 activity assay in vivo and in vitro showed apoptotic effects through increased expression of pro-apoptotic markers (e.g., poly ADP ribose polymerase (PARP); Bcl-2-associated X protein (Bax)), and decreased expression of apoptotic markers (e.g., Bcl-2 and cyclins) [49]. A study used the in vivo tumor xenograft assay and the in vitro MTT and miR-106b ISH assays showed that PCs promote anti-proliferative/invasive effects on NSCLC cells via downregulating miR-106b expression and upregulating cyclin-dependent kinase inhibitor 1A (CDKN1A) mRNA and p21 expression [50].

A few studies on NSCLC cells after treatment with the Cinnamomi Cortex extract PCs showed a significant reduction in nuclear factor-E2-related factor 2(Nrf2) expression, and insulin-like growth factor-1 receptors (IGF-1R) were responsible for induced proliferation [51,52]. Cinnamomi Cortex extract procyanidin C1 exert anti-metastatic activity by suppressing TGF-β-induced EMT in NSCLC cells [53].

Treatment with PCs inhibits hydrogen peroxide (H_2_O_2_)-induced NSCLC cell viability, as shown by reduced ROS and malondialdehyde (MDA) production, hydrogen peroxide(H_2_O_2_)-induced oxidative stress, and promoted the expression of Nrf2 target genes [54]. PCs inhibit proliferation, viability, along with induction of apoptosis and G2/M cell cycle arrest in NSCLC cells. This is triggered by inhibiting the EMT-related molecules (e.g., N-cadherin and vimentin), expression of apoptotic markers (e.g., Bcl-2), and increasing expression of pro-apoptotic markers (e.g., Bax) via downregulating the Janus kinase/signal transducer and activator of transcription3 (JAK2/STAT3) signaling pathway [55].

Treatment with prodelphinidin B-2 3′-O-gallate, a proanthocyanidin gallate, resulted in the upregulation of key transcription factors such as the soluble Fas ligand (sFasL) and membrane-bound Fas ligand (mFasL), which are responsible for the anti-proliferative and apoptotic activities in NSCLC cells [56,57]. Cinnamtannin D1, an A-type procyanidin trimer, from *Rhododendron formosanum* extracts has been found to exhibit autophagic effects on NSCLC cells via inhibition of cellular signaling pathways (e.g., mTOR) [58].

These results suggest that plant-derived natural PCs may play a significant role as anti-NSCLC agents by suppressing proliferation, migration, invasion, viability, metastasis, angiogenesis, and promoting apoptosis/autophagy via inhibition/activation of transcription factors and/or multiple cellular signaling pathways induced by α7nAChR in NSCLC cells. Table 1 highlights the molecular mechanisms of PCs in nicotine-induced NSCLC treatment.

## 4. Anthocyanins in Nicotine-Induced NSCLC Treatment

ACNs-rich extracts from plant-based foods/medicinal plants at various nontoxic concentrations have exerted therapeutic effects against nicotine-induced NSCLC, as demonstrated by several studies. Delphinidin and cyanidin have been shown to inhibit platelet-derived growth factor (PDGF)(AB)-induced VEGF expression in vascular smooth muscle cells through attenuation of the p38 MAPK and c-JUN NH2-terminal kinase (JNK) signaling pathways [59], known as α7nAChR-mediated cascades involved in NSCLC.ACNs extracted from *Vitis coignetiae Pulliat* inhibit the expression of several transcription and growth factors (e.g., MMP2/9 and VEGF) involved in proliferation, angiogenesis, invasion, and migration of NSCLC cells [60]. The extracts from *Morus alba* L. (cyanidin 3-rutinoside and cyanidin 3-glucoside) inhibit the migratory and invasive activities of NSCLC cells [61]. Cyanidin-3-glucoside not only inhibits the proliferation, invasion, and migration, but also induces apoptosis. The mechanism underlying such an effect is associated with inhibiting p53-induced gene 3 (TP53I3) expression in NSCLC cells and the downregulation of cellular signaling pathways (PI3K/Akt/mTOR) involved in NSCLC progression [62].

Delphinidin inhibits tumor growth, angiogenesis, proliferation, and induces apoptosis in NSCLC cells using the in vivo Matrigel plug assay and the in vitro MTT/ELISA assay by upregulating pro-apoptotic expression (e.g., Bax and caspase 3/9), along with downregulating epidermal growth factor receptor (EGFR), cobalt chloride (CoCl2)-induced HIF-1α, Bcl-2, PCNA, cyclin D1, and VEGF mRNA expression via inhibiting of several signaling pathways [63,64].

A combination of five ACN extracts (i.e., delphinidin, peonidin, petunidin, cyanidin, and malvidin) from bilberry and blueberry resulted in inhibited growth and metastasis, and induced apoptosis and cell cycle arrest of NSCLC cells in vitro. The mechanism of action of ACNs involves inhibiting activation of multiple signaling pathways, including TNFα-induced NF-_k_B, Notch, Wnt/β-catenin, and their key transcription factors (i.e., cyclin D1/B1, VEGF, p-ERK, bcl-2, and MMP9). Furthermore, when evaluated with an in vivo model using the in vivo xenograft assay, delphinidin alone, and the ACN mixture, resulted in significantly inhibited growth of H1299 cells [65]. ACNs derived from *Syzygium cumini* L. (known as Indian blackberry) showed significant anti-proliferative effects on NSCLC cells, but the mechanisms for this action have not been observed [66].

These results suggest that ACNs may have a significant role in anti-proliferative, anti-invasive, anti-angiogenic, anti-metastatic, and apoptotic/autophagic effects in NSCLC cells by suppressing the activation of key transcription/growth factors and α7nAChR-mediatedcellular signaling pathways. The molecular mechanisms of ACNs in nicotine-induced NSCLC treatment are summarized in Table 2.

## 5. Combination of Proanthocyanidins and Anthocyanins with Chemotherapeutics/Radiotherapy in Nicotine-Induced NSCLC Treatment

Several studies suggest that PCs and ACNs used in combination with radiotherapy and/or chemotherapy have the ability to modulate the activity of cellular signaling pathways involved in proliferation, inflammation as well as in anti-apoptosis and/or cell cycle arrest in NSCLC cells. Treatment with PCs extracted from Cinnamomi Cortex inhibits Nrf2-mediated proliferation through promoting sensitivity of NSCLC cells to the cytotoxic action of chemotherapeutic drug etoposide and doxorubicin [67]. A recent study using the CCK8 and ELISA assays reported anti-inflammatory and apoptosis-inducing effects of PCs on NSCLC cells when used in combination with ionizing radiation. The results indicated that such combinations inhibit tumor growth in vivo and reduce interferons/interleukins production and increase pro-apoptotic p53 target genes in vitro via upregulating the MAPK signaling pathway [68]. Delphinidin treatment with γ-ionizing radiation (IR) induced autophagy and apoptosis in NSCLC cells by increasing pro-apoptotic expression (e.g., caspase-3 and LC3), and reduced p-ERK1/2 expression via activation of the MAPK signaling pathway [69]. Cyanidin-3-glucoside, but not pelargonidin-3-glucoside, has been recently reported to inhibit the mRNA level of claudin-2 (CLDN2)-induced chemotherapy resistance to doxorubicin through the downregulation of p-Akt and the upregulation of p38 MAPK, leading to apoptosis and necrotic cell death in NSCLC cells [70]. Table 3 summarizes the molecular mechanisms of PCs and ACNs in combination with radiotherapy/chemotherapy in nicotine-induced NSCLC treatment.

## 6. PCs and ACNs as Positive Modulators of Human α7nAChR

Findings of this review suggest that PCs and ACNs exert anti-tumor functions against NSCLC, due to their potential as positive modulators of α7nAChR. Treatment of NSCLC cells with varying concentrations suppresses the growth of these cells by 50% relative to the untreated control (GI_50_). The concentrations used both in vivo and in vitro studies are not associated with gross/visible toxicity. ACNs overdose possesses no significant cytotoxic effects on NSCLC cells in vivo. The cytotoxicities of anti-NSCLC drugs and/or ionizing radiation are significantly improved in the presence of 100–300 µg/mL Cinnamomi Cortex PCs in vitro, 20 µg/mL grape seed PCs in vivo/vitro, and ≤50 µM ACNs in vitro.

PCs vary in the number, configuration, arrangement and substitution of OH groups on the B ring. In general, all PCs have OH groups on C-3, C-5, and C-7 positions, respectively, and on their B rings [22]. The anti-NSCLC activity of PCs may rely on the number of OH groups on their B-ring, and the OH group at C-3 plays a significant role in their significant anti-tumor ability [71]. Procyanidins have five OH groups on positions C3,5,7,4′,5′, with one OH group on the C ring and two OH groups on the A and B rings [22]. Procyanidins may interact with α7nAChR binding site and thus could be considered as a potential target for the treatment of nicotine-induced NSCLC.

The number and position of OH and methoxyl groups on the flavylium B-ring of ACNs may play a significant role in the suppression of α7nAChR and its downstream signaling pathways. The presence of one OH group or methoxy substituents on the B-ring may result in a considerable loss of inhibitory effects in NSCLC cells. Delphinidin, which has three OH moieties at C-3′,4′,5′ of the B-ring, exerts greater inhibitory activity than cyanidin and pelargonidin, which have two and one OH group, respectively. The presence of two OH groups, one methoxyl group in petunidin, and one OH group with methoxy substituents in malvidin and peonidin make them less inhibitory effects on NSCLC cells [25]. Thus, ACNs would be expected to have high levels of effective anti-NSCLC agents in binding to the α7nAChR-mediated signaling pathways, which may result in suppression of the receptor in a concentration-dependent manner.

Understanding conditions associated with nicotine-α7-nAChR signaling might provide an opportunity to explore the therapeutic potential of PCs and ACNs in nicotine-induced NSCLC. Among several types of α subunits, the homomeric α7nAChR has been shown to be the key receptor activating NNK and/or nicotine-mediated cell proliferation, angiogenesis and anti-apoptosis in NSCLC cells in vivo and in vitro [10,72]. High levels of the α7 mRNA levels in squamous cell carcinoma tumors are found in smokers than nonsmokers [73]. NNK has been shown to be a high affinity agonist for α7nAChR in LC cells [74]. Nicotine is converted to NNK via activation of the α7nAChR and β-adrenergic receptor (β-AR) [8]. NNK binds to α7nAChR and β-AR causes activation of NSCLC-promoting signaling pathways [10,72]. NNK induces production of ROS in vitro, which causes oxidative DNA damage in NSCLC cells by activating the Wingless-type protein (Wnt) signaling pathway [75]. NNK or nicotine induces NSCLC cell proliferation, invasion and angiogenesis in vivo and in vitro through α7nAChR with activation of COX-2 expression, leading to an upregulation of PGE_2_, which is implicated in NSCLC development [76,77]. NNK enhances proliferation of NSCLC cells in vitro by activating PCNA expression via PI3K/AKT/Stat3/ERK1/2 signaling pathway activation [78]. In vitro, treatment with nicotine causes an induction effect on α7nAChR, along with significant stimulation of p-ERK1/2 cascade, accompanied by promotes the nAChR-mediated production of neurotransmitter noradrenaline in the brain, which enhances β-AR signaling-mediated DNA synthesis of NSCLC cells, resulting in promoted cell proliferation [79]. Nicotine influences NSCLC biology involve inhibition of apoptosis induced by chemotherapy and radiotherapy, and induction of proliferation and angiogenesis of NSCLC cells. Such effect is mediated by α7nAChR, which stimulates signaling pathways such as PI3K/Akt/mTOR, suppresses of the mitochondrial death pathway or induces of pro-angiogenic factors [80]. The binding of nicotine to the α7nAChRin vivo/vitro upregulates multiple downstream pathways (e.g., MAPK/ERK, Src, JAK, and STAT) and induces the secretion of growth/angiogenic factors (e.g., EGF and VEGF) in NSCLC cells, and such effect could be mediated by inactivation of p53 expression [9]. Binding of nicotine to α7nAChR enhanced voltage-gated Ca^2+^ channels, which result in induction of NSCLC cell angiogenesis by activating of Ca^2+^ influx [10,72]. Nicotine induces NSCLC angiogenesis, migration and invasion in vitro by inhibiting HIF-1α and its downstream target gene VEGF. Such effect is mediated via α7nAChR-mediated Src, Ca+2, PI3K, MAPK/ERK1/2, and mTOR signaling pathways activation [81]. Nicotine promotes NSCLC cell proliferation in vitro via protein arrestin, β1 (ARRB1)-mediated activation of E2F transcription factors 1 (E2F1), and Src and Rb-Raf-1 signaling downstream of α7nAChR [82,83]. Nicotine or NNK induces NSCLC cell proliferation in vivo and in vitro by activating Akt phosphorylation and its related downstream substrates, including GSK-3β, mTOR and NFκB [84]. In vitro, proliferation, invasion and angiogenesis of NSCLC cells are found to be promoted by nicotine via α7nAChR with activation of Signal transducer and activator of transcription1 (STAT1), E2F1, as well as induction of Src, PI3K, and Rb-Raf signaling pathways [85]. The apoptotic family members, including Bcl-XL, c-Myc, and Bcl2, are induced in response to nicotine/NNK treatment in NSCLC cells in vitro via α7nAChR-mediated ERK1/2, Raf1 and MAPKs signaling pathways activation, resulting in suppression of apoptosis [86]. In vivo, nicotine or NNK-induced activation of the PI3K/Akt/mTOR pathway via stimulation of α7nAChR inhibits Bax expression [87]. NNK treatment inhibits miRNAs expression in NSCLC cells in vivo. This is observed through upregulation of cytochrome P450 (CYP) 2A3 expression as a mechanism of such effect [88]. NNK induces NSCLC progression in vitro by altering the expression of mismatch DNA repair (MMR), leading to miRNAs deregulation, particularly miR-422a, miR-155, and miR-21 [89]. NNK induces proliferation and inhibits apoptosis in NSCLC cells in vivo by downregulating miR-124 expression, leading to the activation of Akt, thereby enhancing NSCLC progression [90].

PCs may have the ability to inhibit β-amyloid (Aβ) aggregation [91], which is the key hallmark of NSCLC [92,93]. Aβ is one of the key mechanisms responsible for LC, as shown in increased Aβ isoforms in A549 cells, particularly Aβ40 and Aβ42. Inactivation of p53 was found to increase Aβ40 and Aβ42 levels in A549 cells via upregulation of the PI3K/Akt/NFκB signaling pathway and MMP2/9 expression, known to increase viability and reduce apoptosis in NSCLC cells, suggesting that activation of p53 may be helpful in reducing the levels of Aβ40 and Aβ42 responsible for viability and anti-apoptosis in NSCLC cells [93]. Aβ40 and Aβ42 contain amino acids serine (Ser) and tyrosine (Tyr) (H_6_DSGY_10_ and G_25_SNKG_29_), which are known to be the major toxic Aβ forms. Ser26 and Tyr10 of Aβ peptides have been shown to interact with acetylcholinesterase (AChE), a cholinergic enzyme-mediated neurotransmitter acetylcholine (Ach) associated with amyloid fibril formation in A549 cells, leading to increased cytotoxic response in these cells [94]. Ach produced by AchE from acetyl-coenzyme A (acetyl-CoA) and choline plays a keyrole as paracrine/autocrine growth factor in the lung homeostasis, which binds to nAChRs on the NSCLC cells [95,96]. The expression of AchE has been reported to be reduced in NSCLC cells. The decrease in AchE activity leads to elevate Ach levels in NSCLC cells, which could promote cell proliferation, migration, invasion, angiogenesis, and reduce apoptosis/autophagy through activating Ca^2+^ channels, TGF-β-induced EMT and α7nAChR-mediated signaling pathways such as PI3K/Akt, ERK/MAPK, GSK-3β, and STAT3 [97]. This suggests that Ach may have therapeutic potential in nicotine-induced NSCLC cells by upregulating expression of p53, which promotes apoptosis/autophagy and reduce proliferation and angiogenesis in NSCLC cells. Given that PCs reduce cellular growth and proliferation in NSCLC cells via upregulating p53 expression, targeting such natural compound in treatment regimes might help in downregulating cellular signaling pathways, as the mechanisms involved in Aβ-induced proliferation and anti-apoptosis in NSCLC cells.

Activation of Ach was also found to promote inflammation and pro-inflammatory cytokines in pulmonary tissue [98]. Increased concentrations of pro-inflammatory cytokines (i.e., IL-6, IL-8, IL-1β, and TNF-α) have been reported in NSCLC cells, particularly H460 and A549 cells [99]. The potential mechanism underlying such increases involves activating the α7nAChR-mediated JAK1-dependent STAT3 signaling by receptor tyrosine kinase (RTK) such as VEGFR and mutant-EGFR [100,101]. Therefore, ACNs may have significant anti-inflammatory functions via reducing of Aβ aggregation in NSCLC cells, leading to the modulation of α7nAChR and its downstream signaling pathways.

Previous studies showed that microRNAs (miRs), including miR-205-3p, a class of noncoding RNA molecules, act as potential markers for the progression of NSCLC [102,103]. A recent in vitro experiment has suggested that the Aβ precursor protein-binding family B member 2 (APBB2)-mediated activation of dysregulated miR-205-3p expression in different NSCLC cells, including H460, H1299, 95-D, and A549, resulted in activation of proliferation and inhibition of apoptosis in NSCLC cells [104]. PCs and ACNs may directly bind to Aβ to reduce its aggregation, suggesting that these compounds could be useful in reducing miR-205-3p expression involved in the pathogenesis of NSCLC. PCs and ACNs may thus be useful for their anti-Aβ aggregation activity via reducing miR-205-3p expression, resulting in an inhibition of proliferation and induction of apoptosis in NSCLC cells.

ACNs may bind to the active site of α-L-rhamnosidases (α-R) enzyme, having an inhibitory effect on NSCLC cells by inhibiting the cellular signaling pathways involved in proliferation and anti-apoptosis/autophagy. α-R is involved in ACNs metabolism, which hydrolysis of glycosidic bonds, and thus, may improve the bioavailability as well as the anti-tumor activity of ACNs in nicotine-induced NSCLC cells. For example, α-R enzyme has been demonstrated to catalyze the hydrolysis of cyanidin-3-rutinoside to cyanidin-3-glucoside in black raspberry from *Aspergillus* spp. [105] and in red mulberry (*Morus alba* L.) fruit [106], which are known for their anti-NSCLC activity. Thus, α-R may provide highly biologically active ACNs, which suppress proliferation and induction of apoptosis/autophagy in NSCLC cells.

**Figure 2:**
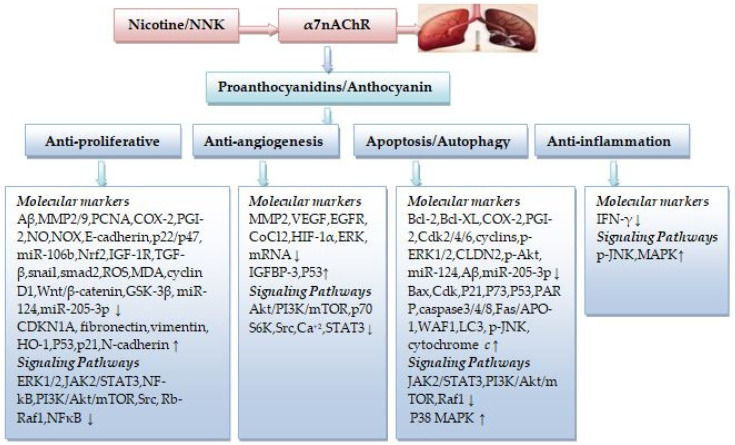
The therapeutic potential of PCs and ACNs in nicotine-induced NSCLC. (↓) decrease, (↑) increase.

Taken together, nicotine-mediated activation of cell proliferation, migration, invasion, angiogenesis, and inhibition of apoptosis in NSCLC cells can be modulated when PCs and ACNs interact with the active site of α7AChR, displaying complex modulation of several transcription/growth factors via signaling pathways involved in α7nAChR activation, suggesting that such compounds may have a significant role in the treatment of NSCLC (Figure 2).

## 7. Limitations

Although the available in vivo and in vitro studies suggest a therapeutic potential of PCs and ACNs in nicotine-induced NSCLC, these studies are used different dosage regimens in the treatment, which questions the reliability of results from these studies. Different doses of PCs and ACNs may affect diverse transcription factors and multiple cellular signaling pathways, and thereby affecting targeted nicotine-induced NSCLC therapy. The optimal effective dose of PCs and ACNs are difficult to determine, as it varied between studies. PCs and ACNs could be effective in nicotine-induced NSCLC treatment if used at concentration of up to 100 μM/µg/mL, but further studies are needed to apply this in vitro anti-NSCLC effects.

## 8. Conclusions

PCs and ACNs are flavonoid pigments with anti-NSCLC effects that exist in various plant-based foods and/or medicinal plants. The potential molecular mechanisms by which PCs and ACNs from plants-based foods and medicinal plants exert anti-tumor activity with therapeutic potential in nicotine-induced NSCLC have not been elucidated. Treatment with PCs and ACNs at various nontoxic concentrations have demonstrated several activities against nicotine-induced NSCLC, including anti-proliferative, anti-migration, anti-metastatic, anti-invasive, anti-angiogenic, and apoptosis/autophagy induction. PC-rich extracts from grape seed and/or Cinnamomi Cortex used in combination with irradiation/chemotherapy may have useful anti-proliferative, anti-inflammatory and apoptotic efficacy against nicotine-induced NSCLC. Delphinidin and cyanidin may promote apoptotic/autophagic activity by enhancing the chemosensitivity and/or radiosensitivity of NSCLC cells.

Nicotine and/or NNK may contribute to NSCLC development by promoting cell proliferation, migration, invasion, angiogenesis, and inhibiting apoptosis. Such effects may occur through α7nAChR-mediated signaling pathways activation. PCs and ACNs may act as selective agonists of α7nAChR, where they can inhibit the activation of α7nAChR in NSCLC cells. PCs and ACNs may directly bind to α7nAChR, suggesting that these compounds could be useful in modulating multiple transcription/growth factors and signaling pathways. PCs may act as anti-NSCLC agents through inhibiting excessive accumulation of Aβ involved in nicotine-induced NSCLC. In addition, the α-R enzyme involved in deglycosylation of ACNs in plant-based foods may play a key therapeutic role in nicotine-induced NSCLC, as they are involved in the anti-proliferative and apoptotic/autophagic activities against NSCLC cells. Therefore, plant-based foods and/or medicinal plants-derived PCs and ACNs may have therapeutic efficacy as anti-NSCLC medicinal agents in smokers due to their anti-proliferative, anti-angiogenic, anti-migration, anti-invasive, anti-inflammation, anti-metastatic, and apoptosis/autophagy activities, but further studies are needed to better understand the underlying mechanisms.

## Figures and Tables

**Table 1:** The molecular mechanisms of PCs in nicotine-induced NSCLC treatment.

Study Type	NSCLC Cell Type	Extract/Compound	Concentrations	Activity	Mechanisms of Action	Reference
In vitro	DMS114	Cranberry presscake	200–300 µmol/L	Anti-proliferative, apoptosis	NA	[40]
In vitro	H460	Cranberry (*Vaccinium macrocarpon*)	20–80 µg/mL	Anti-proliferative	MMP2, MMP9↓	[41]
In vitro	H460	Cranberry (*Vaccinium macrocarpon*)	50 µg/mL	Apoptosis, cell cycle arrest	P21, P73, PARP, cytochrome *c*, caspase3/4/8↑ Bcl-2↓	[42]
In vitro/vivo	A549	Grape seed	100 µg/mL (in vitro) 30 mg PC/kg bodyweight (in vivo)	Inhibition of tumor angiogenesis	MMP2↓	[43]
In vitro/vivo	A549, H1299	Grape seed	60, 80 µg/mL (in vitro) Administration of PCs (0.1%, 0.2%, and 0.5%, bodyweight) (in vivo)	Anti-proliferative, anti-angiogenic, apoptosis	PCNA↓ IGFBP-3↑	[44]
In vitro/vivo	H157, H226, H460, H1299, A549	Grape seed	20, 40, and 60 µg/mL (in vitro) Administration of PCs (0.5%, bodyweight) (in vivo)	Anti-proliferative, apoptosis	COX-2, PGI-2↓	[45]
In vitro	A549, H1299	Grape seed	10, 20, 40, and 60 µg/mL	Anti-migration	NO, L-NAME, MAPK, ERK1/2↓	[46]
In vitro	A549	Grape seed	6 µg/mL	Anti-proliferative, apoptosis	caspase 3, PTGIS/PGI2↑	[47]
In vitro	A549, H1299, H460	Grape seed	20 and 40 µg/mL	Anti-migration	E-cadherin, NOX, p22/p47(phox)↓ N-cadherin, fibronectin, vimentin↑	[48]
In vitro/vivo	A549, H1299	Grape seed	20, 40, and 60 µg/mL (in vitro) 50, 100, and 200 mg PC/kg bodyweight (in vivo)	Apoptosis	G1arrest, Bax, caspases-3/9, Cdki, PARP↑ Bcl-2,Bcl-xl, Cdk2/4/6, cyclins↓	[49]
In vitro/vivo	A549	Grape seed	45 µg/mL (in vitro) 112 mg PC/kg bodyweight (in vivo)	Anti-proliferative, anti-invasive	CDKN1A, p21↑, miR-106b↓	[50]
In vitro	A549	Cinnamomi Cortex	2.5 µg/mL	Inhibition of cell viability and proliferation	Nrf2↓	[51]
In vitro	A549	Cinnamomi Cortex	10 µg/mL	Inhibition of cell proliferation	Nrf2, IGF-1R↓	[52]
In vitro	A549	Cinnamomi Cortex	12.5, 25, 50, and 100 µg/mL	Anti-metastatic	TGF-β, snail, E-cadherin, smad2↓	[53]
In vitro	A549	PCs	≥100 mg/L	Inhibition of cell viability	ROS, MDA, Nrf2↓ HO-1, NQO1, TXNRD1, glutathione, catalase, superoxide dismutase↑	[54]
In vitro	A549	PCs	12.5, 25, 50, 100, and 200 µM	Inhibition of cell viability and proliferation, apoptosis, cell cycle arrest	N-cadherin, vimentin, Bcl-2, MMP2/9, JAK2/STAT3↓, Bax↑	[55]
In vitro	A549	Green tea leaf	1, 5, 10, 20 µM	Anti-proliferative, apoptosis, cell cycle arrest	P21, P53, Fas/sFasL, Fas/APO-1↑	[56]
In vitro	A549	*Myrica rubra*	0.5, 2.5, 5, and 10 µM	Anti-proliferative, apoptosis, cell cycle arrest	Fas/APO-1, P21/WAF1, P53, Fas/sFasL↑	[57]
In vitro	A549, H460	*Rhododendron formosanum*	125, 150, and 175 µM	Autophagy	Akt/mTOR↓	[58]

(↑) increase; (↓) decrease; NA: not mentioned.

**Table 2:** The molecular mechanisms of ACNs in nicotine-induced NSCLC treatment.

Study Type	NSCLC Cell Type	Extract/Compound	Concentrations	Activity	Mechanisms of Action	Reference
In vitro	A549	*Vitis coignetiae Pulliat*	200 µg/mL	Anti-proliferative, anti-invasive, anti-angiogenic, anti-migration	MMP2/9, cyclin D1, C-myc, COX-2, VEGF↓	[60]
In vitro	A549	*Morus alba* L.	25, 50, and 100 µM	Anti-migration, anti-invasive	MMP2, c-Jun, C-fos, NF-kB↓	[61]
In vitro	A549, H1299	Cyanidin-3-glucoside	5, 10, 20, 40, and 80 µM	Anti-proliferative, anti-migration, anti-invasive, apoptosis	TP53I3 andPI3K/Akt/mTOR↓	[62]
In vitro/vivo	A549, H441, SK-MES-1	Delphinidin	5–100 µM (in vitro) 1, 2 mg PC/kg bodyweight (in vivo)	Inhibition of tumor growth, anti-proliferative, anti-angiogenic, apoptosis	EGFR, Bcl-2, PCNA, cyclin D1, VEGFA, Akt/PI3K/MAPK↓ Bax, caspase-3/9↑	[63]
In vitro/vivo	A549	Delphinidin	10, 20, and 40 µM (in vitro) 80 µM (in vivo)	Anti-angiogenic	EGF, CoCl2, HIF-1α, ERK, VEGF mRNA, Akt/mTOR/PI3K/p70S6K↓	[64]
In vitro/vivo	A549, H1299	Bilberry and blueberry	3.125–12.5 µM (in vitro) 1.5 mg PC/kg bodyweight (in vivo)	Anti-metastatic, anti-invasive, apoptosis, cell cyclearrest	TNFα-induced NF-_k_B, Notch, Wnt/β-catenin, cyclinD1/B1, VEGF, p-ERK, bcl-2, MMP9 ↓	[65]
In vitro	A549	*Syzygium cumini* L.	2.5, 5, 10, 20, and 25 µM	Anti-proliferative	NA	[66]

(↑) increase, (↓) decrease, NA; not mentioned.

**Table 3:** The molecular mechanisms of PCs and ACNs in combination with radiotherapy/chemotherapy in nicotine-induced NSCLC treatment.

Study Type	NSCLC Cell Type	Extract/Compound	Concentrations	Activity	Mechanisms of Action	Reference
In vitro	A549	Cinnamomi Cortex	100–300 µg/mL	Anti-proliferative	Nrf2↓	[67]
In vitro/In vivo	A549	Grape seed	20 µg/mL (in vitro) 2 mg PC/kg bodyweight (in vivo)	Anti-inflammation, apoptosis	ROS, IL-6, IFN-γ↓ P53, p-JNK, MAPK↑	[68]
In vitro	A549	Delphinidin	10, 20, and 50 µM	Apoptosis, autophagy	p-ERK1/2↓, caspase-3, LC3, p-JNK, MAPK ↑	[69]
In vitro	A549	Cyanidin-3-glucoside	1, 10, 20, and 50 µM	Apoptosis, autophagy	CLDN2, p-Akt↓, P38 MAPK↑	[70]

(↑) increase; (↓) decrease.
